# Strongly subradiant states in planar atomic arrays

**DOI:** 10.1515/nanoph-2023-0624

**Published:** 2024-01-31

**Authors:** Ilya A. Volkov, Nikita A. Ustimenko, Danil F. Kornovan, Alexandra S. Sheremet, Roman S. Savelev, Mihail I. Petrov

**Affiliations:** Department of Physics, ITMO University, Saint-Petersburg, Russia

**Keywords:** atomic lattices, subradiance, collective quantum excitations, external coupling, flat bands

## Abstract

The optically trapped ensembles of atoms provide a versatile platform for storing and coherent manipulation of quantum information. However, efficient realization of quantum information processing requires long-lived quantum states protected from the decoherence e.g. via spontaneous emission. Here, we theoretically study collective dipolar oscillations in finite planar arrays of quantum emitters in free space and analyze mechanisms that govern the emergence of strongly subradiant collective states. We demonstrate that the external coupling between the collective states associated with the symmetry of the array and with the quasi-flat dispersion of the corresponding infinite lattice plays a crucial role in the boost of their radiative lifetime. We show that among different regular arrangements of the atoms the square atomic arrays support eigenstates with minimal radiative losses $\propto N_{\text{tot}}^{-5}$ scaled with the total number of atoms *N*_tot_.

## 1 Introduction

In the recent years, a significant progress has been achieved in the development of artificial quantum interfaces based on arrays of cold atoms [[Bibr j_nanoph-2023-0624_ref_001] [Bibr j_nanoph-2023-0624_ref_002] [Bibr j_nanoph-2023-0624_ref_003]. Such ensembles of qubits being ordered with advanced optical manipulation methods in the free space [2], [4] or in the vicinity of nanophotonic structures [5], [6], [7] have already demonstrated potential in storing, processing and transmitting quantum information [8]. However, implementation of these technological achievements in practical devices requires solving a number of fundamental problems such as storing and protecting quantum information from decoherence.

One of the possible ways to overcome these problems is to suppress spontaneous emission of quantum emitters, which can be achieved in the *subradiant* collective states in the ensembles of qubits. Superradiance and subradiance effects have been examined in atomic systems [9], [10], [11], in nanophotonics [12], [13] and in radio antennas as well [14], [15]. The start of studying these phenomena was given by the pioneering theoretical work of Dicke [16], which was followed by experiments with small atomic clouds [17] and a couple of ions [18]. Recently, such states have been largely explored in one-dimensional atomic chains coupled to a waveguide [19], [20], [21] and in large atomic clouds in free space [22]. However, the advantages of two-dimensional (2D) arrays such as methods of precise optical positioning with a high filling factor [1], convenience of transmittance and reflection spectra measurements [4], [23], possible convenient application of Rydberg blockade [24], and many others, resulted in outstanding progress in optical manipulation over qubit states [25]. Moreover, a number of novel theoretical approaches were proposed for photon manipulations with atomic lattices [26], [27], [28], [29].

**Figure 1:**
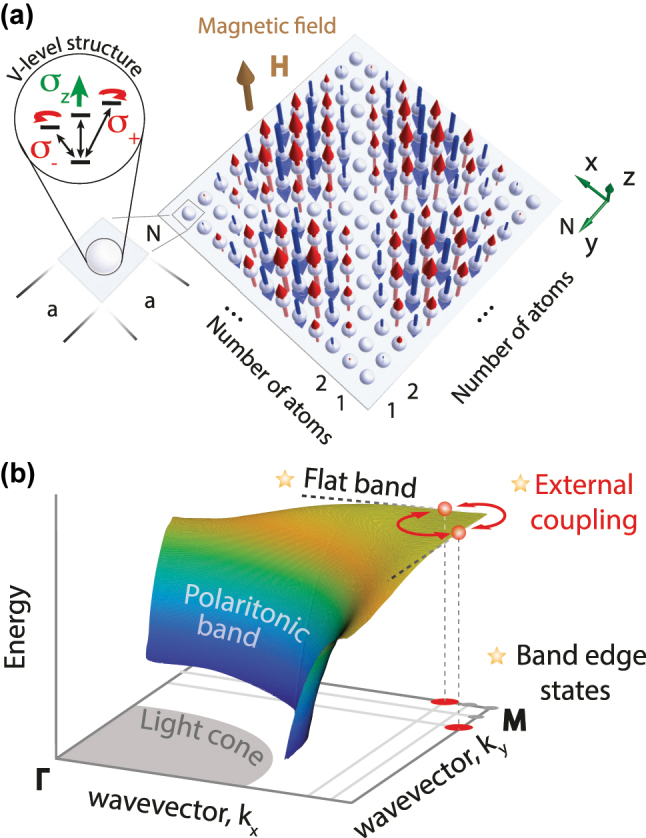
Schematic of the subradiant states formation in a regular planar atomic array. (a) A two-dimensional finite square regular lattice of *N* × *N* atoms with *σ*_*z*_ transition, separated by distance *a*. The distribution of the highly non-radiative mode of *B*_2_ symmetry is shown with arrows. (b) Polaritonic band diagram of the infinite dipolar lattice. The main mechanisms responsible for the formation of non-radiative states are shown with stars.

The studies of the long-living states in atomic arrays were in the center of several works, revealing two main types of the subradiant states for 2D systems in free space. The first type of the states in the square 2D lattice with out-of-plane dipole moment orientation and in-phase coherence (near the Γ-point of the Brillouin zone [BZ], see Figure 1) was shown to have radiative losses proportional to $N_{\text{tot}}^{-1}$ [30], [31], where *N*_tot_ is the total number of atoms in the array. Such states fall in the type of bound states in continuum extensively studied recently in photonics [32], [33], [34], [35], and have been further a subject for investigation in atomic arrays [31], [36], [37]. The second type of subradiant states inherits its non-radiative properties from the guided waves of the infinite lattice appearing below the light cone. Similar states in 1D chains were shown to exhibit much faster size dependence of the radiative losses than the bound states in continuum, namely $N_{\text{tot}}^{-3}$ , $N_{\text{tot}}^{-5}$ or $N_{\text{tot}}^{-7}$ [20], [38], [39], depending on the period of the chain. While in the 2D case such states were studied only in the Ref. [40] revealing $N_{\text{tot}}^{-3}$ scaling law for the specific square geometry of the array.

In this work, we consider planar finite arrays of two-level atoms arranged in different regular structures with the focus on the square atomic arrays schematically shown in Figure 1(a). We identify the main factors affecting the radiative losses suppression in such structures, including interference of the out-of-phase oscillating neighbor dipoles in the far zone, external coupling of the states associated with the symmetry of the structure, and “accidental” external coupling that emerges due to quasi-flat polaritonic band dispersion of the corresponding infinite lattice, see Figure 1(b). We establish the conditions under which these mechanisms are present and demonstrate the interplay between them on the example of the square array. We show that the radiative decay rate of the most subradiant states in square arrays decrease as fast as $\propto N_{\text{tot}}^{-3}$ or $\propto N_{\text{tot}}^{-5}$ (*N*_tot_ = *N* ^2^ is the total number of atoms in the array), which leads to very long lifetimes of the eigenstates even in compact arrays. Moreover, there exist optimal geometrical parameters providing resonant increase of radiative lifetime by a few orders. We also compare lifetimes of the most subradiant states for different lattice geometries, showing advantage of square lattice. In addition, we demonstrate the possibility of selective excitation of the most subradiant states by vector Bessel beams with a suitably chosen orbital angular momenta.

## 2 General formalism

Let us consider an array of identical two-level atoms in free space with a transition frequency *ω*_0_ (transition wavelength *λ*_0_) and spontaneous decay rate $\Gamma_{0}=\omega_{0}^{3}|d{|}^{2}/\left(3\pi \hbar \varepsilon_{0}c^{3}\right)$ , where *ɛ*_0_ is vacuum permeability and *d* is the amplitude of the transition dipole moment. We assume that the atoms are separated by the distance more than $∼0.1\lambda_{0}$ , *λ*_0_ = 2*πc*/*ω*_0_, thus neglecting the Casimir interaction between atoms in the ground state due to its very fast spatial decay $∼1/r^{6}$ [41]. Consequently, the coupling between the atoms is determined by the dipole–dipole interaction, governed by the electromagnetic free space Green’s tensor **G**(**R**, *ω*) [42].

To describe such interaction, we introduce dipole moment operator of the *i*th atom as ${\hat{d}}_{i}=d{\hat{\sigma }}_{i}+d^{*}{\hat{\sigma }}_{i}^{†}$ , where ${\hat{\sigma }}_{i}=|g_{i}〉〈e_{i}|$ , ${\hat{\sigma }}_{i}^{†}=|e_{i}〉〈g_{i}|$ are the atomic lowering and rising operators describing the transition from the excited state |*e*_*i*_⟩ to the ground |*g*_*i*_⟩ state of the *i*th atom and vice versa, respectively. The interaction Hamiltonian of the system ${\hat{H}}_{\text{int}}$ , describing both re-scaterring processes governed by dipole–dipole interaction between *N*_tot_ atoms and spontaneous emission into the free space, can be obtained as the total interaction energy between dipole moments and electric field: ${\hat{H}}_{\text{eff}}=-\sum_{i=1}^{N_{\text{tot}}}{\hat{d}}_{i}\hat{E}\left(r_{i}\right)$ , where $\hat{E}\left(r_{i}\right)$ is the electric field operator at the position **r**_*i*_. Exploiting classical principle of electric field superposition and the connection between the electric field and Green’s tensor [40], [43], [44], one can directly obtain the effective Hamiltonian [45], [46]:

(1) $$\begin{array}{ll} {\hat{H}}_{\text{eff}} & =\hbar \left(\omega_{0}-\frac{i}{2}\Gamma_{0}\right)\overset{N_{\text{tot}}}{\underset{i=1}{\sum }}{\hat{\sigma }}_{i}^{†}{\hat{\sigma }}_{i}\\ & \;\;\;-\underset{i\neq j}{\sum }\frac{3\pi \hbar \Gamma_{0}}{k_{0}}e_{d}^{*}\cdot G\left(R_{ij}\right)\cdot e_{d}\;{\hat{\sigma }}_{i}^{†}{\hat{\sigma }}_{j}, \end{array}$$

where **e**_**d**_ = **d**/*d* is the unit vector of the atomic transition dipole moment. In Eq. (1), the first term describes the coupling of atoms to free space, while the second one describes the atom–atom coupling. The above equation is obtained within the Markovian approximation by neglecting the frequency dependence of the Green’s tensor, **G**(**R**, *ω*) ≈ **G**(**R**, *ω*_0_), which is justified by a very narrow resonance of a single atom Γ_0_ ≪ *ω*_0_.

The eigenvalues and eigenstates of the Hamiltonian (1) define the complex eigenfrequencies *ω*_*j*_ − *i*Γ_*j*_/2 and eigenfunctions Ψ^(*j*)^ of the collective dipolar excitations, respectively. Real and imaginary parts of the eigenfrequency correspond to the frequency and radiative decay rate of the *j*th collective eigenstate. For convenience throughout the paper we define the frequency with respect to that of a single atom, Δ*ω*_*j*_ = *ω*_*j*_ − *ω*_0_.

Normally, in the absence of the external magnetic field, atoms are spherically symmetric, and the simplest model of such an atom is the one with triply degenerate excited state (*S* ↔ *P* transition). In the presence of the *z*-oriented magnetic field (normal to the *x* − *y* plane of the array, see Figure 1(a)) the states of an atom can be spectrally separated into three states depending on the polarization of the transition dipole moment, *σ*_−_, *σ*_+_, or *σ*_*z*_. Consequently, the collective eigenstates of the planar arrays of such atoms can be considered separately for different polarizations of the atomic transitions.

## 3 Square atomic array

It is known that the radiative losses are mostly suppressed in symmetric structures, which possess fewer radiation channels than non-symmetric ones. In this section, we focus on one of the most simple and high-symmetry case of 2D atomic array *N* × *N* arranged is a square lattice with a period *a*. Such illustrative example allows us to demonstrate the competition between different mechanisms of the radiative loss suppression.

### 3.1 Classification of the collective eigenstates

The eigenstates of the finite lattices can be classified and characterized based on their distribution in the quasi-reciprocal space [19], [47], [48] which can be done by expanding the dipole moments distribution of the *j*th eigenstate Ψ^(*j*)^ in the standing (Bloch) waves basis:

(2) $$\begin{array}{ll} \Psi_{n_{x},n_{y}}^{\left(j\right)} & =\underset{m_{x},m_{y}}{\sum }c_{m_{x},m_{y}}^{\left(j\right)}\psi_{n_{x},n_{y}}^{\left(m_{x},m_{y}\right)}\\ & =\frac{2}{N+1}\underset{m_{x},m_{y}}{\sum }c_{m_{x},m_{y}}^{\left(j\right)}\sin\left(q_{0}m_{x}n_{x}\right)\sin\left(q_{0}m_{y}n_{y}\right), \end{array}$$

where $c_{m_{x},m_{y}}^{\left(j\right)}$ are the expansion coefficients, *q*_0_ = *π*/(*N* + 1) is the discretization quasi-wavevector, *m*_*x*,*y*_ = 1…*N* are the numbers of the basis state in the quasi-reciprocal space, and *n*_*x*,*y*_ = 1…*N* are the atoms positions in the array in the real space. Such basis is straight-forwardly constructed as a tensor product of the two sets of standing waves in *x* and *y* directions. Two indices, *m*_*x*_ and *m*_*y*_, that specify the basis state $\psi^{\left(m_{x},m_{y}\right)}$ , show that the standing wave distribution of the dipole moments changes the sign  *m*_*x*_ − 1 and *m*_*y*_ − 1 times in *x* and *y* directions, respectively. For example, the basis state that is closest to the corner of the BZ (*M*-point in Figure 1(b)) in the reciprocal space is characterized by the indices *m*_*x*_ = *m*_*y*_ = *N*.

**Figure 2:**
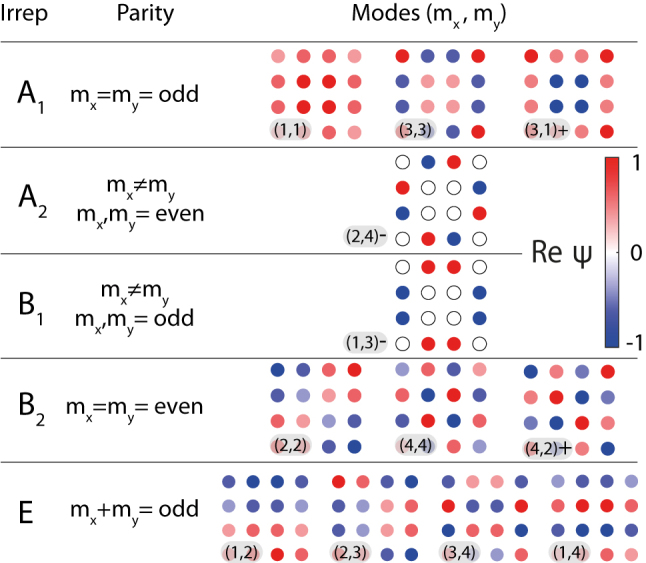
Symmetry classification of the basis functions $\psi^{\left(m_{x},m_{y}\right)}$ for a finite square lattice. Classification is based on the irreducible representations and normalized quasi-wavenumbers *m*_*x*_ and *m*_*y*_ (see Eq. (2)). Wavefunctions are plotted for the case of 4 × 4 lattice, color shows real part of the wavefunction amplitude at each lattice site.

Alternative natural classification of the eigenstates is based on their symmetry properties. The square array belongs to the *C*_4*v*_ point symmetry group and thus the eigenstates Ψ^(*j*)^ should transform according to one of the irreducible representations (irreps) *A*_1_, *A*_2_, *B*_1_, *B*_2_, *E*. However, the basis given by Eq. (2) does not account for the specific symmetry of the square array. Therefore, not all of the basis states $\psi^{\left(m_{x},m_{y}\right)}$ fall into the irreps of the *C*_4*v*_ point symmetry group and, thus, should be symmetrised. Then, the following correspondence between the basis states and irreps, summarized in Figure 2, can be established. The basis states with odd value of (*m*_*x*_ + *m*_*y*_) transform according to *E* irreducible representation and have “azimuthal numbers” ±1 (or their linear combination), i.e. phases of the neighbor corner dipoles differ by *π*/2. The basis states with *m*_*x*_ = *m*_*y*_ transform according to *A*_1_ (*B*_2_) irreducible representation if *m*_*x*,*y*_ is odd (even), and have “azimuthal numbers” 0 (2), i.e. phases of the neighbor corner dipoles are the same (differ by *π*). If *m*_*x*_ ≠ *m*_*y*_ and (*m*_*x*_ + *m*_*y*_) is even the mode $\psi^{\left(m_{1},m_{2}\right)}$ hybridises with the mode $\psi^{\left(m_{2},m_{1}\right)}$ and they form two linear combinations:

(3) $$\psi^{{\left(m_{1},m_{2}\right)}^{\pm }}=\frac{1}{\sqrt{2}}\left(\psi^{\left(m_{1},m_{2}\right)}\pm \psi^{\left(m_{2},m_{1}\right)}\right).$$

Antisymmetric modes transform according to *A*_2_ or *B*_1_, while symmetric modes transform according to *A*_1_ or *B*_2_ irrep.

### 3.2 Mechanism of the radiation suppression

If the eigenstate Ψ^(*j*)^ of the atomic array is mainly characterized by a single dominant contribution of a basis state $\psi^{\left(m_{x},m_{y}\right)}$ , its radiative losses Γ_*j*_ are qualitatively determined by its *location in the BZ*, which defines the efficiency of the destructive interference between the neighbouring dipoles in the array. The losses are typically weaker for the states that are closer to the *M*-point in the reciprocal space, i.e. when *m*_*x*_, *m*_*y*_ → *N*, due to the larger mismatch between the wavevector of such state and the free-space wavevector.

The second factor affecting the radiative losses is associated with the symmetry of a given eigenstate, which is determined by the *symmetry of the finite array*. For instance, Bloch waves propagating in an infinite square lattice in directions near the *M*-point and symmetric with respect to the Γ*M*-direction are degenerate owing to the symmetry of the lattice, see Figure 1. However, in a finite array the degeneracy is lifted due to the *external coupling* of these waves according to Friedrich–Wintgen mechanism [49] leading to the formation of symmetric and antisymmetric states. The latter ones transform according to *A*_2_ or *B*_1_ irreps and are antisymmetric with respect to the diagonal vertical planes of symmetry *n*_*x*_ = ±*n*_*y*_, i.e. the excitation of the corner dipole moments of such states is necessarily zero. Due to the suppressed scattering from the sharp edges of the structure, the radiative losses of these states can substantially decrease [48], [50], [51]. On the other hand, symmetric modes that transform according to *A*_1_ or *B*_2_ with non-zero corner dipole moments are characterized with the increased losses.

Additional mechanism of the losses suppression may appear due to the external coupling of the eigenstates that is not related to the symmetry of the infinite lattice or the finite array, but rather to the accidental degeneracy of the Bloch waves in the corresponding infinite lattice. As we show further, such regime can be realised for small enough periods, when the lattice dispersion becomes *quasi-flat*. In this case, two (or typically several) basis states interact via radiation continuum resulting in an additional suppression or increase of their radiative losses. The overall radiative losses of an eigenstate are then determined as a result of the competition between these three mechanisms of loss suppression. In order to determine which of the eigenstates exhibits minimal losses for a given size and period of the structure, we perform straight-forward numerical calculations and the results are presented in the next subsection.

### 3.3 Numerical calculations of the finite array eigenstates

**Figure 3:**
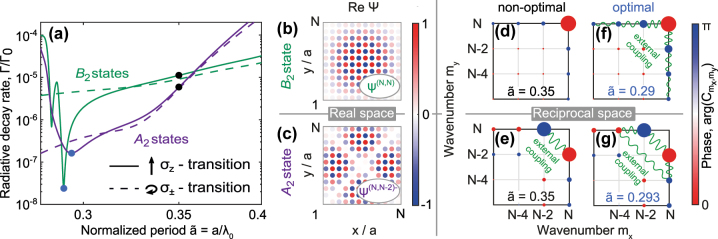
Characteristics of the subradiant states in 2D square array. (a) Normalized radiative decay rate of two most subradiant eigenstates corresponding to *B*_2_ (green curves) and *A*_2_ (violet curves) symmetry in 12 × 12 square array as a function of the normalized period $\tilde{a}$ . (b, c) Real part of the wavefunction plotted in real space for the states shown in (a) with green and violet solid curves, respectively. (d–g) Expansion of the eigenstates, Eq. (2), shown in (a) with (d, e) black and (f, g) blue points; size of the circles indicate the amplitude of the corresponding harmonics; wavy lines illustrate the coupling between different harmonics.

In Figure 3(a) we show numerically calculated emission rates, normalized by the emission rate of a single atom, Γ/Γ_0_ for two collective states of the 12 × 12 square array as a function of the normalized period $\tilde{a}=a/\lambda_{0}$ . These states are characterized by the minimal emission rates among the others. Solid lines correspond to the *σ*_*z*_ dipoles, while the dashed lines to the *σ*_+_ or *σ*_−_ ones. One can observe that for periods $\tilde{a}≳0.32$ , the radiative losses monotonously increase with the decrease of the period. Standing wave decomposition Eq. (2) performed in this range of periods reveals that all states are characterized by the dominant contribution either of a single basis state or two basis states in the symmetrized form, Eq. (3). The numerical analysis revealed that the most subradiant states (in the array with even *N*) transform according to either *B*_2_ or to *A*_2_ irrep. The distribution of the wavefunction of the *σ*_*z*_ polarized states in real space is shown in Figure 3(b) and (c), while its representation in the reciprocal space, i.e. the decomposition Eq. (2), for the period $\tilde{a}=0.4$ is shown in Figure 3(d) and (e). The distribution for the *σ*_±_ looks similar. As expected, the *B*_2_ state is the closest one to the *M*-point and it is well approximated by the *ψ* ^(*N*,*N*)^ basis function. For this state, dipole moments of the neighbouring atoms are almost out-of-phase, which leads to their efficient destructive interference.

However, the radiative losses of such state are minimal only for the periods $\tilde{a}≳0.36$ . For smaller periods, the *A*_2_ state, which is the second closest to the *M*-point, becomes more subradiant. This state has the dominant components *m*_*x*_ = *N*, *m*_*y*_ = *N* − 2 and *m*_*x*_ = *N* − 2, *m*_*y*_ = *N* in the antisymmetric combination $\psi^{{\left(N,N-2\right)}^{-}}$ , Eq. (3) (see also Figure 1). According to the symmetry properties of the irrep *A*_2_, the dipole moments of such state are equal to zero on diagonals. It leads to small probability that the excitation resides on the corners of the square, see Figure 3(c), resulting in suppressed scattering from sharp edges and consequently weak radiative losses of such state in the most range of the periods.

Such antisymmetric subradiant states can also be viewed as the result of the external coupling between two eigenstates of the rectangular array that appears under stretching/squeezing the lattice to the square one [52], see also Supplementary Materials, Section A for the details. Consequently, the mechanism of the symmetry-induced external coupling is very sensitive to the deformation of the lattice: in a rectangular lattice the efficiency of the coupling drops very fast, resulting in the substantially increased losses of the $\psi^{{\left(N,N-2\right)}^{-}}$ state even for very small detunings from the square geometry of the order of 1 %. On the other hand, the state *ψ* ^(*N*,*N*)^ survives under strong deformation of the lattice up to tens of percents, since it is not associated with the specific symmetry of the structure.

For even smaller lattice periods, the decay rate as a function of the period exhibits a minimum around $\tilde{a}\approx 0.3$ for both *A*_2_ and *B*_2_ states and for *σ*_*z*_ polarization, see Figure 3(a). At these minima the wavefunctions of the states in the real space remain similar to those in Figure 3(b) and (c). However, the expansion in the reciprocal space, Eq. (2), shown in Figure 3(f) and (g), reveals that the amplitudes of other harmonics are substantially increased. The interaction between these harmonics leads to additional suppression of radiative losses near the period $\tilde{a}\approx 0.3$ . At the same time, the calculations for the *σ*_±_ dipole transitions, dashed curves in Figure 3(a), show that radiative losses are monotonous function of the period in this case and no resonant suppression of the losses via external coupling mechanism is observed.

### 3.4 Infinite atomic lattices

**Figure 4:**
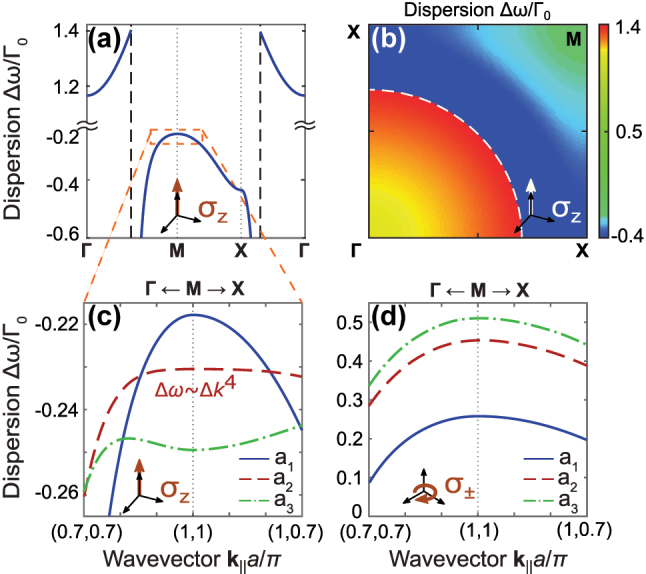
Dispersion of 2D square atomic lattice. (a, b) Dispersion of an infinite square lattice of *σ*_*z*_ polarized atoms for the period $\tilde{a}=0.35$ (a) along high symmetry path and (b) in the whole BZ. (c, d) Dispersion near the *M* point for periods ${\tilde{a}}_{1}=0.35$ (blue curves), ${\tilde{a}}_{2}=0.294$ (red curves) and ${\tilde{a}}_{3}=0.28$ (green curves) for (c) *σ*_*z*_ polarized atoms and (d) *σ*_±_ polarized atoms.

Appearance of the external coupling near $\tilde{a}∼0.3$ for *σ*_*z*_ polarized dipoles and its absence for the *σ*_±_ ones can be linked to the behaviour of the infinite lattice dispersion of the polaritonic states near the *M*-point. To illustrate this, we have performed calculations of the band diagrams of the square lattice for both polarizations of the dipole transitions and for different periods, using the analytical Floquet summation approach [43], [53], see details of the calculations in the Supplementary Materials, Section B. First, we consider the most interesting case of the *z*-oriented dipoles. In Figure 4(a), we plot the dispersion along the main direction in the first BZ. Figure 4(b) complements the panel Figure 4(a) with the 2D colorplot in the whole BZ. Above the light cone, shown with the black dashed lines in Figure 4(a) and with white dashed curve in Figure 4(b), the Bloch states are leaky and the eigenstates of the corresponding finite arrays are characterized with large radiative losses. On the other hand, the modes below the light line are guided (the eigenfrequency is purely real) and the eigenstates of the corresponding finite arrays are subradiant.

As it was shown in the previous subsection, the eigenstates of the finite arrays that are closest to the *M*-point, are the most subradiant due to the largest mismatch with the wavevectors of the free space. One can observe in Figure 4(c) that the dispersion near the *M*-point turns out to be qualitatively different for different periods. For the large periods $\tilde{a}≳0.3$ , the interaction between the dipoles is rather weak, so that the dispersion is parabolic. For the lattice period near $\tilde{a}=0.294$ , the dispersion exhibits an inflection point and becomes quartic, as shown with the red curve in Figure 4(c). For even smaller periods $\tilde{a}≲0.294$ , the dispersion becomes nonmonotonous near the *M*-point along the Γ*M* direction (green curve in Figure 4(c)), which explains the external coupling of the several states as now their energies match with each other, analogously to the one-dimensional case [38]. In contrast, dispersion in the case of *σ*_+,−_ polarized dipole transition, shown in Figure 4(d) with dashed curves, remains monotonic near the *M*-point for any subwavelength period. This explains the absence of the external coupling in finite arrays for this polarization.

## 4 Discussion

### 4.1 Variation of the radiative losses with the size of arrays with different geometries

**Figure 5:**
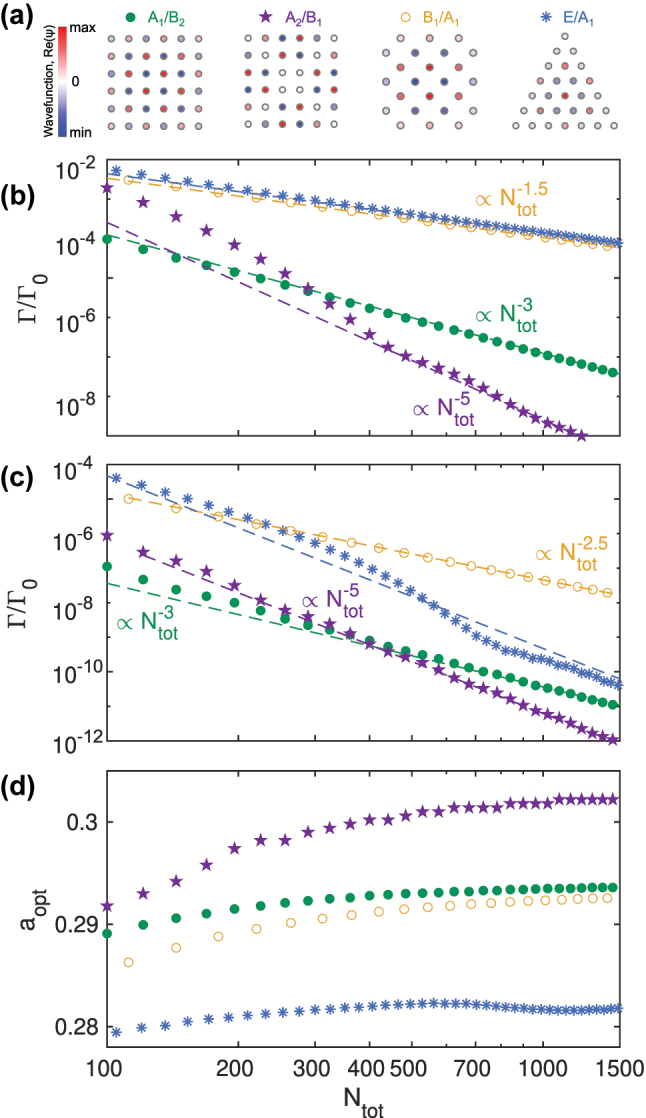
Scaling of the radiative losses with the size of the array. (a) Array geometries and wavefunctions of the most subradiant eigenstates. (b, c) Radiative losses of subradiant states as a function of the total number of atoms in the array *N*_s_ for different geometries for (b) fixed distance between the neighbor atoms $\tilde{a}=0.4$ , (c) optimal period for each *N*_tot_, shown in panel (d). Dashed lines indicate corresponding polynomial functions.

The mechanisms determining the formation of the subradiant states in atomic arrays that were shown on the example of the square geometry have rather general nature and are present in various configuration of atomic ensembles. Calculations performed for a few other main geometries of the regular atomic arrays such as rotated square, triangle, hexagon (see Supplementary Materials, Section C for the details) have revealed that the square geometry is preferable for the state lifetime. In Figure 5(b) we show decay rates of the four characteristic eigenstates, corresponding to different array geometries shown in Figure 5(a), as a function of the total number of the atoms *N*_tot_, calculated for the non-optimal period $\tilde{a}=0.4$ when there is no accidental external coupling between the eigenstates caused by the quasi-flat dispersion. The polarization of the dipoles was chosen to be along the *z* direction. The scaling of the *A*_2_/*B*_1_ state as $N_{\text{tot}}^{-5}\equiv N^{-10}$ in a square array, shown with purple pentagrams in Figure 5(b), turns out to be unique. For instance, diagonal square or triangle arrays exhibit only $\propto N_{\text{tot}}^{-1.5}$ scaling laws providing much shorter lifetimes than the square geometry even for rather small *N*_tot_.

Another type of the eigenstate of the square array, *A*_1_/*B*_2_ states with the dominant *ψ* ^(*N*,*N*)^ contribution, exhibit only $\propto N_{\text{tot}}^{-3}\equiv N^{-6}$ dependence of the decay rate. Such difference in the scaling laws for this particular geometry can be qualitatively explained in a simple manner, as elaborated in the Supplementary Materials, Section D. Despite the slower decrease of the decay rate for *A*_1_/*B*_2_ states, such asymptotic dependence is valid only for large number of atoms, while it does not necessarily reflect the difference between the radiative losses for a particular *N*_tot_. One can observe in Figure 5(b), that the antisymmetric state becomes the most subradiant only for *N*_tot_ ≳ 300 for a given period $\tilde{a}=0.4$ .

In Figure 5(c), we plot the calculated decay rates for the optimal periods ${\tilde{a}}_{\text{opt}}$ , for which the losses are minimal (i.e. period is optimized for each value of *N*_tot_), as indicated in Figure 5(d). Here, the presence of external coupling leads to a change of the scaling laws in some geometries while remaining the same $\propto N_{\text{tot}}^{-3}$ and $\propto N_{\text{tot}}^{-5}$ in the square geometry. This contrasts with the one-dimensional case where interaction between two standing waves leads to the decrease of the asymptotic behaviour of the subradiant state decay rate from ∝*N* ^−3^ to ∝*N* ^−7^ [20], [38]. Although, e.g. in a triangle geometry decrease of the decay rate also becomes close to $\propto N_{\text{tot}}^{-5}$ , the absolute values of the decay rate still remain minimal for the *A*_2_/*B*_1_ states of the square geometry.

Note that the presented analysis on the asymptotic behavior of radiative losses was performed in the ideal case of a perfect periodicity of the array and the absence of various decoherence effects inevitably presented in any realistic system of coupled quantum emitters. For instance, disorder effects can significantly influence the radiative losses [54], especially in the case of subradiant states in large systems. While the signatures of subradiant states themselves can be detected in realistic systems, the observation of effects related to the quasi-flat dispersion [20] and the symmetry of states is currently quite challenging. At the same time, it was recently shown for atomic clouds, that subradiance is quite robust to thermal decoherence [55].

### 4.2 Excitation of the subradiant states

In order to demonstrate the possibility of excitation of the strongly subradiant states appearing for the *σ*_*z*_ polarization, we consider the scattering of vector Bessel beams [56], [57] by a square atomic array. The use of such specific excitation field has two underlying reasons. First, we consider the states characterized by large wavevectors. The use of Bessel beams, which can have arbitrary angular momentum, allows for partially matching the azimuthal component of the wavevector of the incident field to that of the eigenstate. Second, Bessel beams have non-zero longitudinal electric field, which allows for efficient excitation of the *σ*_*z*_ polarized states. The considered highly inhomogeneous incident field may excite various modes of the lattice with different efficiency, therefore we have optimized orbital number of the beam *m* to maximize the overlap between external electric field and the desired collective state, see the Supplementary Materials, Section E for details of the scattering calculations. We need to stress here that while the full description of the quantum dynamics of the considered system, accounting for various atomic decoherence mechanisms, requires approach based a master equation for the density matrix [46], [58], here we use semiclassical simulations (see Supplementary Materials, Section E), which give exactly the same results for spontaneous emission decoherence.

**Figure 6:**
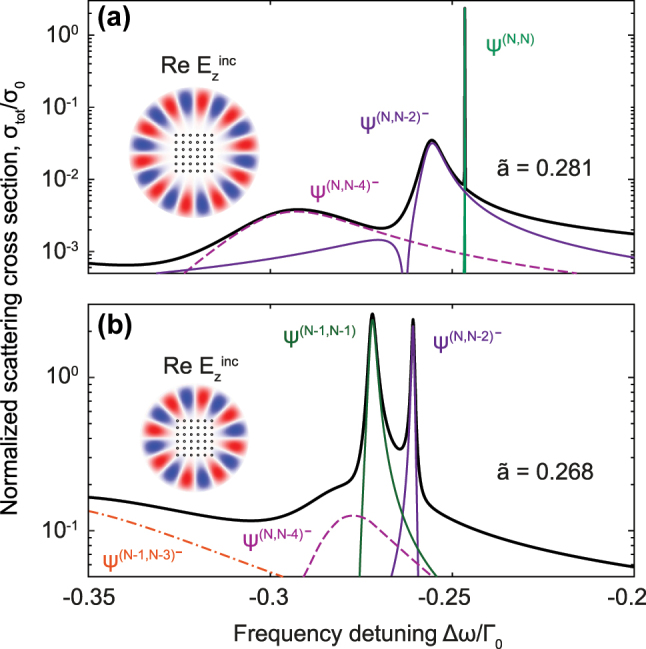
Normalized scattering cross section spectra of 6 × 6 square atomic lattice irradiated by Bessel beam with (a) azimuthal number *m* = 9 and period $\tilde{a}=0.281$ and (b) *m* = 7 and $\tilde{a}=0.268$ . Solid black curves correspond to the total scattering cross section spectra, while colored curves correspond to different eigenstates with dominant contributions of basis states indicated in the plots. Insets show distributions of *z* component of the incident electric field.

In Figure 6, we show numerically calculated scattering cross-section spectra of 6 × 6 square atomic array irradiated by normally incident vector Bessel beam with a spin *s* = 1 due to circular polarization, and orbital numbers *l* = 7 or *l* = 9 [57]. Total cross-section consists of various peaks matching eigenmodes of the lattice characterized with different dominant basis states. The parameters of the beams were chosen to achieve the maximum coupling with the eigenstates with dominant contribution of basis states $\psi^{{\left(N,N-2\right)}^{-}}$ and *ψ* ^(*N*,*N*)^, respectively. Lattice periods correspond to optimal periods in terms of radiative losses of these states, $\tilde{a}=0.268$ and $\tilde{a}=0.281$ in the case of 6 × 6 array. Cross-section spectra are normalized by a maximum value of a single atom cross-section *σ*_0_.

As one can see in the insets of Figure 6, the optimal incident field profile barely overlaps the atomic array from the outside, setting *π* phase shift between neighboring atoms at the edges with almost no influence at internal atoms. Therefore, a total angular momentum of the beam *l* + *s* matches to either a half of total number of edge atoms 2(*N* − 1), see Figure 6(a), or the same number but excluding corner atoms 2(*N* − 2), see Figure 6(b). In the first case, the beam is capable to excite the *B*_2_ state with a dominant *ψ* ^(*N*,*N*)^ contribution. In the second case, where the field at the corners is absent, it is optimal to excite *A*_2_ state with a dominant $\psi^{{\left(N,N-2\right)}^{-}}$ contribution. Thereby, a fine tuning of the incident beam frequency and angular momentum allows for selectively exciting the desired long-living eigenmode of the array.

### 4.3 Relation to high quality modes in nanophotonic cavities

Importantly, the generality of the reported results is also underlined by the tight connection to the research area of engineering of planar dielectric nanophotonic cavities, such as homogeneous dielectric cavities [52], [[Bibr j_nanoph-2023-0624_ref_059] [Bibr j_nanoph-2023-0624_ref_060] [Bibr j_nanoph-2023-0624_ref_061] [Bibr j_nanoph-2023-0624_ref_062] [Bibr j_nanoph-2023-0624_ref_063] and periodic or quasi-periodic nanostructured cavities [65], [66].

The resonators made of homogeneous dielectric are expected to have the highest *Q*-factors for spherical or cylindrical geometries, which exhibit the highest symmetry. However, the utilized fabrication methods or proposed technological applications sometimes demand the use of the cavities of the other shapes, e.g. cubes, squares, hexagons etc., which were also shown to support rather high *Q*-factors and have potential in development of micro and nanolasers [52], [67], [68]. The dispersion of homogeneous dielectric is, however, quasi-linear and the external coupling due to quasi-flat band in this case is not possible. Unlike the homogeneous cavitites, the dispersion in the nanostructured ones, i.e. photonic-crystal or nanoparticle cavities, is more tunable. Consequently, quasi-flat and nonmonotonous bands in such structures result in the quality factor boost in the finite arrays [66]. By proper translating all mechanisms of the subradiant states formation reported in this work for the dipolar arrays onto the nanophotonic platform could lead to the development of the novel designs of the compact optical micro and nanocavities.

## 5 Summary

To summarize, we have studied main factors that define the radiative loss suppression in finite-size planar atomic arrays. We have shown that the square arrays support eigenstates with minimal losses among different regular arrangements of the atoms, that scale with the number of atoms as $\propto N_{\text{tot}}^{-5}$ . The two distinct types of external coupling associated with the symmetry of the array and with the quasi-flat dispersion of the corresponding infinite array play the dominant role in the strong boost of the radiative lifetime of such states. We believe, our findings might provide useful insight in designing of the cold atoms based qubit arrays for storing and coherent manipulation of quantum information.

## Supplementary Material

Supplementary Material Details
