# Supplementary material for: Strongly subradiant states in planar atomic arrays
Source: Nanophotonics. 2024 Jan 31;13(3):289–98. doi: 10.1515/nanoph-2023-0624 (PMC11502005; doi:10.1515/nanoph-2023-0624)
Supplement: Supplementary file 1 — Supplementary Material Details [file j_nanoph-2023-0624_suppl_001.pdf]

# Strongly subradiant states in planar atomic arrays: Supplementary Material

## CONTENTS

A. Effect of deformation of the square array on the eigenstates decay rates 2

B. Calculation of the infinite lattice dispersion 3

C. Subradiant states in atomic arrays with different geometry 5

D. Qualitative explanation of the losses scaling with the size of the square array 7

E. Details of scattering calculations 9

References 10

## A. EFFECT OF DEFORMATION OF THE SQUARE ARRAY ON THE EIGENSTATES DECAY RATES

In this section, we analyze sensitivity of the radiative decay rate of the two most subradiant states in the square atomic array (see Fig. 3 in the main text) to the deformation of the lattice. To illustrate this, we have calculated decay rates of the two most subradiant states in the square $12 \times 12$ array arranged in a rectangular lattice with periods $\tilde{a}_x, \tilde{a}_y$ as a function of the ratio of the periods $a_y/a_x$. The results of the calculations for the period $\tilde{a}_x = 0.31$ are shown in Figs. S1(a,b) with dotted curves. We should note that in the case of rectangular lattice, which belongs to the point symmetry group $C_{2v}$, all the eigenstates are divided into four symmetry types depending on their transformation with respect to the two vertical symmetry planes. On the other hand, in the case of square lattice (with equal number of atoms along $x$ and $y$ directions), which belongs to the $C_{4v}$ point symmetry group, there are five type of states. Unlike the square lattice, in the rectangular lattice all of the states with the dominant contribution of the $\psi^{(N,N)}$, $\psi^{(N,N-2)}$ or $\psi^{(N-2,N)}$ harmonic (see Eq. (2) in the main text) fall into the same irreducible representation. In Figs. S1(a,b) eigenstates with the dominant contribution of the $\psi^{(N,N)}$ and $\psi^{(N-2,N)}$ harmonics, respectively, are shown.

For large enough periods and not very large anisotropy of the lattice, the state with dominant contribution of $\psi^{(N,N)}$ harmonic does not interact with any other state upon the change of the $a_y/a_x$ ratio around $a_y/a_x = 1$, since this state is not associated with the specific symmetry of the square lattice. Consequently, the radiative decay rate in Fig. S1(a) shown with the point curve changes only slightly even for relatively large detunings of the $a_y/a_x$ from one up to several percents. On the other hand, the state with dominant contribution of $\psi^{(N-2,N)}$ harmonic hybridizes with the $\psi^{(N,N-2)}$ harmonic when the periods are close to be equal, i.e. the lattice is almost square. Such symmetry-induced external coupling leads to the strong suppression of losses of such state in the square lattice. Reciprocally, one can say, that the antisymmetric states in the square lattice are rather sensitive to the deformation, resulting in the substantially increased losses even for a very small detunings $\Delta(a_y/a_x) \lesssim 1\%$ from the square geometry, see Fig. S1(b), point curve.

For even larger detuning ($a_y/a_x \approx 0.9$ ($\approx 0.94$) for $B_2$ ($A_2$) state for the given period $\tilde{a}_x = 0.31$) there appears additional external coupling between with the $\psi^{(N,N-2)}$ harmonic caused by the quasi-flat band dispersion of the lattice. This also leads to substantial suppression

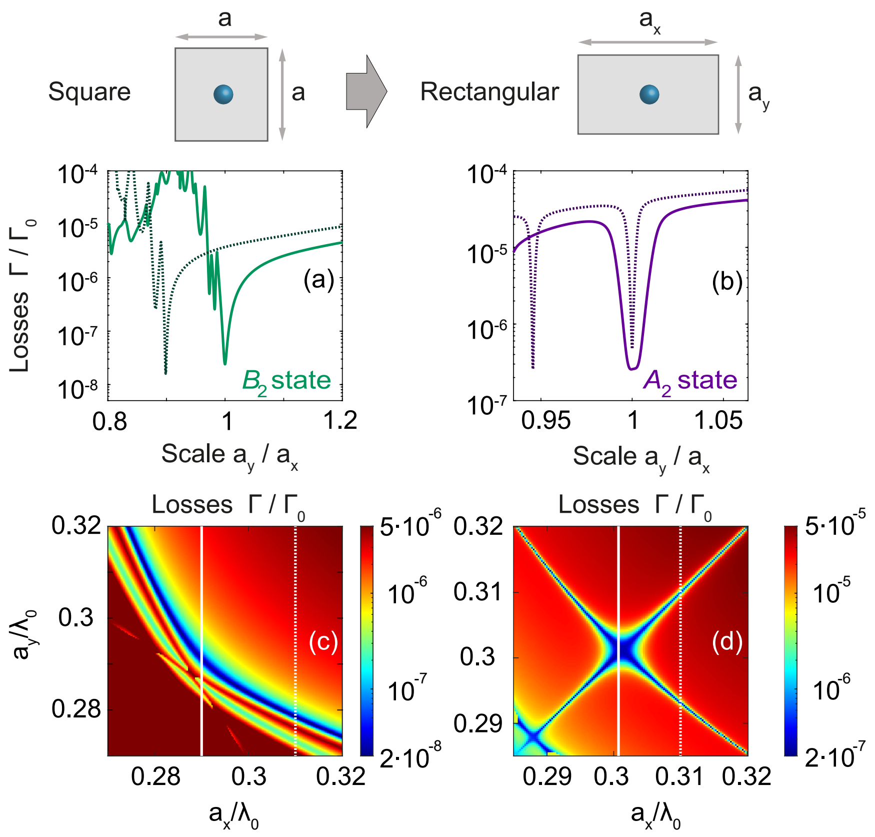


Figure S1. Normalized radiative decay rate of most subradiant $B_2$ (a,c) and $A_2$ (b,d) states in rectangular $12 \times 12$ array. In (a) and (b) period $\tilde{a}_x$ along $x$-axes is fixed whereas period $\tilde{a}_y$ along $y$-axis is changing. In colormaps (c) and (d) both periods $\tilde{a}_x$ and $\tilde{a}_y$ are varied. In (a) and (c) solid lines correspond to $\tilde{a}_x = 0.29$, dotted lines — to $\tilde{a}_x = 0.31$, whereas in (b) and (d) solid lines correspond to $\tilde{a}_x = 0.301$, dotted lines — to $\tilde{a}_x = 0.31$.

of losses with the same level as in the square lattice with optimal period, shown with solid curves in Figs. S1(a,b). Fig. S1(c,d) summarizes the effects of stretching/squeezing of the square lattice on the decay rates of the subradiant states.

## B. CALCULATION OF THE INFINITE LATTICE DISPERSION

Here, we provide the details of the calculations of the dispersion of the various two-dimensional dipole lattices in a free space. According to the Bloch theorem, it is possible to present a eigenstate of an arbitrary Bravais lattice as $|\psi_{\mathbf{k}}\rangle = \sum_j e^{i\mathbf{k}\cdot\mathbf{R}_j}\sigma_j|0\rangle$, where $\mathbf{R}_j$ is a position vector of atom $j = 1\ldots\infty$. Such Bloch state is fully defined by the quasi-

momentum $\mathbf{k}$, which can always be chosen to be within the first Brillouin zone. Plugging a Bloch state into the Hamiltonian, Eq. (1) from the main text, for $N \to \infty$ and multiplying both sides of it by $\langle \psi^\dagger_{\mathbf{k}}|$, we obtain the following general relations for the eigenfrequency and the decay rate [1]:

$$\begin{aligned} \frac{\Delta\omega(\mathbf{k})}{\Gamma_0} &= -\frac{3\pi}{k_0}\mathrm{Re}[C(\mathbf{k})], \\ \frac{\Gamma(\mathbf{k})}{\Gamma_0} &= 1 - \frac{6\pi}{k_0}\mathrm{Im}[C(\mathbf{k})], \end{aligned} \tag{S1}$$

where $\mathbf{R}_{ij} = \mathbf{R}_j - \mathbf{R}_i$, $C(\mathbf{k}) = \sum_{i\neq j} \mathbf{e}^*_{\mathbf{d}} \cdot \mathbf{G}(\mathbf{R}_{ij}) \cdot \mathbf{e}_{\mathbf{d}}\ e^{i\mathbf{k}\cdot\mathbf{R}_{ij}}$, $\mathbf{e}_{\mathbf{d}}$ is the unit vector parallel to the dipole moment transition, and $\mathbf{G}(\mathbf{R}_{ij})$ is the electromagnetic Green's tensor of a free space [2]:

$$\begin{aligned} \mathbf{G}(\mathbf{R},\omega_0) = &\frac{ik_0}{6\pi}\delta^{(3)}(\mathbf{R})\hat{\mathbf{I}} + \\ &+ \frac{e^{ik_0R}}{4\pi R}\left[\left(1 + \frac{i}{k_0R} - \frac{1}{k_0^2R^2})\hat{\mathbf{I}} + (1 - \frac{3i}{k_0R} - \frac{3}{k_0^2R^2}\right)\frac{\mathbf{R}\otimes\mathbf{R}}{R^2}\right], \end{aligned} \tag{S2}$$

where $k_0 = \omega_0/c$ is a vacuum wavevector and $\hat{\mathbf{I}}$ is the unit dyadic.

Note that infinite series in Eqs. (S1) have convergence issues due to the farfield term $e^{ik_0R}/R$ in Eq. (S2). To overcome this problem, we exploit the Poisson summation approach, which was fully described for a rectangular lattice consisted of in-plane dipoles in Ref. [3]. For a case of the $z$-polarised dipoles, one can step-by-step follow the derivation procedure in the Ref. [3], with a straight-forward substitution of the Green's tensor component $G_{zz}$ instead of $G_{xx}$ and obtain a dipole sum:

$$\begin{aligned} C(\mathbf{k}) = &\frac{1}{4\pi a_x}\sum_{\varepsilon=\pm1}\left[\mathrm{Li}_1(e^{ia_x(k_0+\varepsilon k_x)}) + \frac{i}{k_0a_x}\mathrm{Li}_2(e^{ia_x(k_0+\varepsilon k_x)}) - \frac{1}{k_0^2a_x^2}\mathrm{Li}_3(e^{ia_x(k_0+\varepsilon k_x)})\right] + \\ &+ \frac{1}{\pi a_x k_0^2}\sum_{Re[p_m]\neq0}\sum_{n=1}^{+\infty}\left[k_0^2K_0(p_ma_yn) - \frac{p_m}{a_yn}K_1(p_ma_yn)\right]\cos(k_yna_y) + \\ &+ \frac{1}{2\pi a_xk_0^2}\sum_{Re[p_m]=0}\left\{(k_0^2 + \frac{p_m^2}{2})\left[\ln(\frac{|p_m|a_y}{4\pi}) + i\frac{\pi}{2} + \gamma\right] - \frac{p_m^2}{4} - \frac{k_y^2}{2} - \frac{\pi^2}{3a_y^2} + \right. \\ &+ \frac{\zeta(3)a_y^2}{32\pi^2}\left[4k_0^2(2k_y^2 - p_m^2) + p_m^2(4k_y^2 - p_m^2)\right] + \frac{\pi}{a_y}\left[ik_z^{(m,0)} + \frac{k_0^2}{ik_z^{(m,0)}}\right] + \\ &+ \sum_{n=1}^{+\infty}\frac{\pi}{a_y}\left[\frac{q^2}{ik_z^{(m,n)}} + \frac{k_0^2}{ik_z^{(m,-n)}} + ik_z^{(m,n)} + ik_z^{(m,-n)}\right] - \frac{4\pi^2n}{a_y^2} - \frac{k_0^2}{n} - \frac{p_m^2}{2n} - \\ &\left. - \frac{a_y^2}{32\pi^2n^3}\left[4k_0^2(2k_y^2 - p_m^2) + p_m^2(4k_y^2 - p_m^2)\right]\right\}, \end{aligned} \tag{S3}$$

where:

$$p_m = \sqrt{(k_x^{(m)})^2 - k_0^2}, \quad k_x^{(m)} = k_x + \frac{2\pi m}{a_x},$$

$$ik_z^{(m,n)} = \sqrt{(k_x^{(m)})^2 + (k_y^{(n)})^2 - k_0^2}, \quad k_y^{(n)} = k_y + \frac{2\pi n}{a_y}.$$

In Eq. (S3), $\mathrm{Li}_n$ is a polylogarithm of order $n$, and $\mathrm{K}_m$ is a Macdonald function of order $m$. Also, here we use specific mathematical constants, such as Euler's constant $\gamma \approx 0.577$ and value of Riemann zeta function $\zeta(3) \approx 1.202$.

To obtain analogous dipole sum for the dipole with circular polarized transition moment in a rectangular lattice, one should substitute $\mathbf{e_d}$ with the $\mathbf{e}_\pm = \frac{1}{\sqrt{2}}(\mathbf{e_x} \pm i\mathbf{e_y})$, which results in the change of $G_{zz}$ to the $\frac{1}{2}(G_{xx} + G_{yy})$. Therefore, one can directly use find expressions (A19, A22) obtained in Ref. [3] for $G_{xx}$ ($x$-oriented dipoles), then apply it for $G_{yy}$ ($y$-oriented dipoles) with a permutation of $x$ and $y$. Finally, taking a half sum for two dipole sums, one may get the final answer.

The obtained infinite series can be accurately truncated and calculated numerically due to fast convergence of order $1/n^4$ [3]. After plugging the obtained dipole sums into Eq. (S1), taking certain parameters of a square lattice, one may get dispersion dependencies $\Delta\omega(\mathbf{k})$ in the main text, see Fig. 1(a) and Fig. 4.

## C. SUBRADIANT STATES IN ATOMIC ARRAYS WITH DIFFERENT GEOMETRY

We have numericaly studied subradiant states in regular arrays of different geometry arranged in square or hexagonal lattices. Here, we present the results of numerical simulations for the considered cases: (i) square array arranged in square lattice cut out along the unit vectors, (ii) square array arranged in diagonal square lattice, (iii)/(iv) triangle/hexagonal array cut out of the hexagonal lattice. Such arrays belong to $C_{4v}$, $C_{4v}$, $C_{3v}$ and $C_{6v}$, respectively. The schematics of the structures are given in the Fig. S2. Note that the geometry (ii) can be viewed as the square cut out along the diagonals of the simple square lattice.

The considered square and hexagonal infinite lattices both exhibit quasiflat-band dispersion for the period $\tilde{a} \approx 0.3$ for the case of $z$ oriented dipole transition moments of the emitters (not shown here). Consequently, we distinguish two different regimes similarly to the main text. First one, when the normalized lattice period $\tilde{a}$ is larger than $\approx 0.3$; in

| | | | | | | |
|---|---|---|---|---|---|---|
| **Geometry of the structure** | | Square array/ square lattice | | Square array/ centered-square lattice | Triangle array/ hexagonal lattice | Hexagonal array/ hexagonal lattice |
| | | 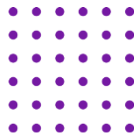 | | 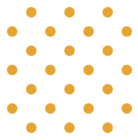 | 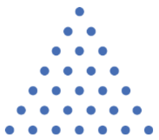 | 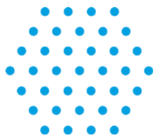 |
| **Symmetry of the structure** | | $C_{4v}$ | | $C_{4v}$ | $C_{3v}$ | $C_{6v}$ |
| **Wavefunction, Re(ψ)** 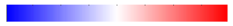 min 0 max | | 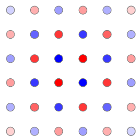 | 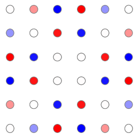 | 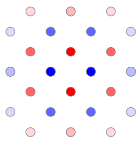 | 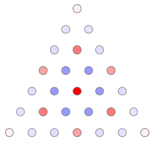 | 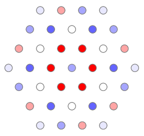 |
| **Symmetry of the mode** | | $A_1/B_2$ | $A_2/B_1$ | $B_1/A_1$ | $E/A_1$ | $A_1/B_2$ |
| **Fixed distance between the atoms a=0.4λ$_0$** | **Scaling of Γ with size** | $N_{tot}^{-3}$ | $N_{tot}^{-5}$ | $N_{tot}^{-1.5}$ | $N_{tot}^{-1.5}$ | $N_{tot}^{-1.5}$ |
| **Optimal distance a$_{opt}$** | **Scaling of Γ with size** | $N_{tot}^{-3}$ | $N_{tot}^{-5}$ | $N_{tot}^{-2.5}$ | $N_{tot}^{-5}$ | $N_{tot}^{-1.5}$..$N_{tot}^{-5}$ |

Figure S2. Table containing the schematics of the considered arrays and their main characteristics.

further simulations we fix the period as $\tilde{a} = 0.4$ in this regime. The second one, corresponds to $\tilde{a} \lesssim 0.3$, when additional external coupling due to quasiflat-band dispersion appears. In this regime, in the calculations we optimize the period for each value of $N_{tot}$ to achieve the minimal decay rate.

We numerically calculated the radiative decay rate as a function of $N_{tot}$ for all eigenstates of all considered arrays, and for two above-mentioned regimes. For each geometry we have identified the symmetry of the most subradiant eigenstates (in the limit of large number of atoms in array $N_{tot}$) and plotted the wavefunction of this state in an array of few atoms for the illustrative purposes, see Fig. S2, third row. The radiative decay rate of the most subradiant states as a function of $N_{tot}$ is shown in Fig. S3(a). The calculations revealed that for the large $N_{tot}$ the states with the $A_2/B_1$ symmetry in the square array are the most subradiant ones among the considered geometries for any period. For large periods they exhibit unique $N_{tot}^{-5}$ decrease of the radiative losses with the size of the array, while other geometries show much slower $N_{tot}^{-1.5}$ dependence.

For smaller periods the size dependencies remain the same in the square arrays, while they change in the other geometries, Fig. S3(b). Moreover, some of the states start to exhibit slightly non-monotonic dependence, see e.g. $A_2$ mode of the diagonal square array in Fig. S3(b). Surprisingly, the triangle array with the sharpest corners supports eigenstates with dependence close to $N_{tot}^{(-5)}$. For not so large number of atoms $N \lesssim 100$, however, the size scaling rate becomes even less specific and the decay rate of the hexagon eigenstates can slightly exceed those of the square array ones.

## D. QUALITATIVE EXPLANATION OF THE LOSSES SCALING WITH THE SIZE OF THE SQUARE ARRAY

The difference between the scaling of the radiative losses with the size of the square array — $N_{tot}^{-3}$ and $N_{tot}^{-5}$ ($N_{tot} = N^2$ is the number of atoms in the array) for $A_1/B_2$ and $A_2/B_1$ states, respectively — can be intuitively explained as follows. As we have shown in Fig. 3(d,e) in the main text, for the non-optimal periods $\tilde{a} \gtrsim 0.3$ two considered eigenstates can be approximated as $\psi^{(N,N)}$ ($A_1/B_2$ state) and $\psi^{(N-2,N)^-}$ ($A_2/B_1$ state) basis states. Since these basis states correspond to the guided waves in the corresponding infinite array, it is natural to expect that the for large size of the array the radiative losses will be determined by the intensity of the wave at the sharp edges of the array [4], i.e. by square amplitudes of the dipole moments near the corners. For the $\psi^{(N,N)}$ state the corner dipole moment from Eq. 2 in the main text reads:

$$\psi_{1,1}^{(N,N)} = \frac{2}{\pi} q_0 \sin^2(q_0) \propto q_0^3, \tag{S4}$$

where the approximation is done for small $q_0 = \pi/(N+1)$ or large $N$. Since in this case $q_0 \propto N^{-1}$, the scaling of the square amplitude of the corner dipole is $|\psi_{\text{corner}}^{(N,N)}|^2 \propto N^{-6} = N_{tot}^{-3}$ in agreement with the numerical simulations.

The corner dipole moment of the $\psi^{(N-2,N)^-}$ state is necessarily zero, since it is antisymmetric with respect to the diagonals of the square. Therefore, we consider the dipole that is next to the corner one:

$$\psi_{1,2}^{(N-2,N)^-} = -\frac{\sqrt{2}}{\pi} q_0 [\sin(q_0)\sin(6q_0) - \sin(3q_0)\sin(2q_0)] \propto q_0^5. \tag{S5}$$

Due to specific symmetry of such state the first term in the expansion $\propto q_0^3$ cancels out

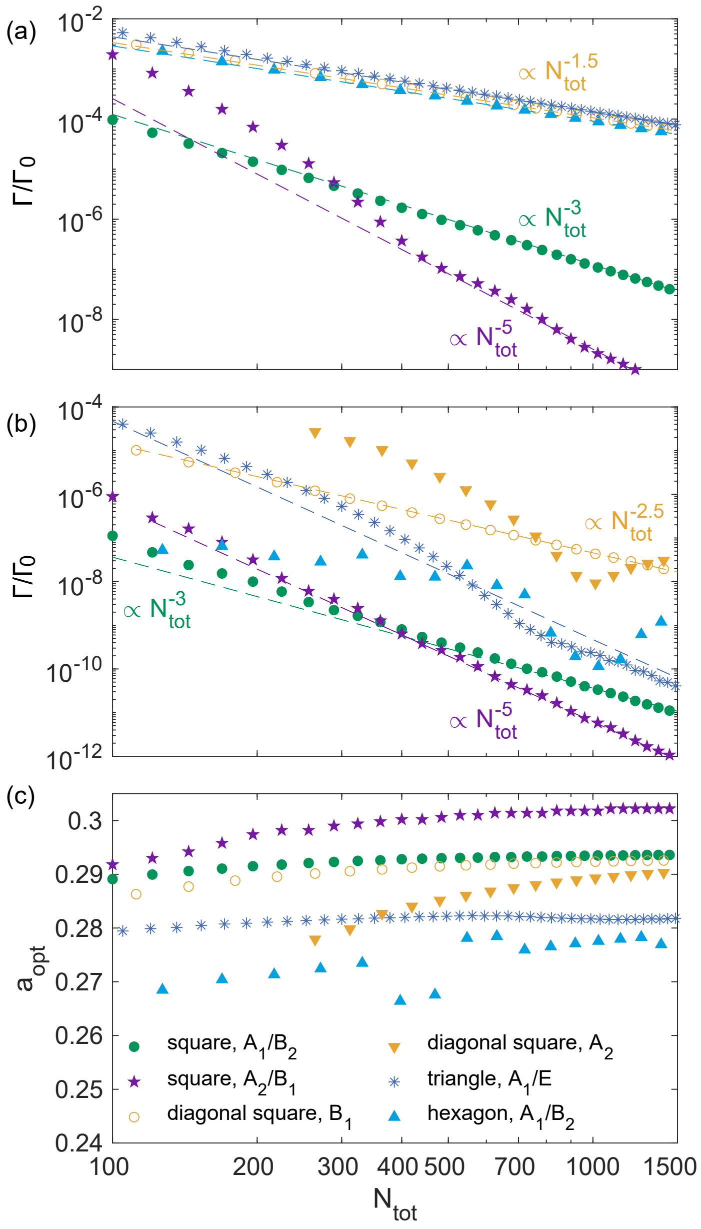


Figure S3. (a,b) Radiative losses of subradiant states as a function of the total number of atoms in the array $N_{tot}$ for different geometries illustrated in Fig. S2 for (a) fixed distance between the neighbor atoms $\tilde{a} = 0.4$, (b) optimal period for each $N_{tot}$, shown in panel (c). Dashed lines indicate corresponding polynomial functions. The legend in (c) is valid for all panels.

and the scaling with $N$ becomes $|\psi_{\text{corner}}^{(N-2,N)^-}|^2 \propto N^{-10} = N_{tot}^{-5}$, again in agreement with the numerical simulations.

## E. DETAILS OF SCATTERING CALCULATIONS

We perform the scattering calculations in a semiclassical manner, where the incident wave with a given spatial distribution $\mathbf{E}_0(\mathbf{r})$ polarizes atoms, which response is described with a semiclassical atomic polarizability $\alpha(\omega) = -\frac{3\pi}{2k_0^2}\frac{\Gamma_0/2}{\omega-\omega_0+i\Gamma_0/2}$. As a result of re-scattering of the photons by the atoms, a dipole moment of the $j$-th atom can be found as:

$$\mathbf{d}^{(j)} = \alpha(\omega)\mathbf{E}_0(\mathbf{r_j}) + \sum_{k\neq j} 4\pi k_0^2 \mathbf{G}(\mathbf{R_{jk}})\mathbf{d}^{(k)}. \tag{S6}$$

By solving the system of linear equations Eq. (S6) for all the atoms, we find the distribution of the dipole moments induced by the incident field, which further allows to obtain the total extinction (scattering) cross-section by using the optical theorem [5]:

$$\sigma_{tot} = \frac{4\pi k_0}{|\mathbf{E}_0(\mathbf{r})|^2}\sum_j \mathrm{Im}\,\mathbf{d}^{(j)}\mathbf{E}_0^*(\mathbf{r}_j). \tag{S7}$$

To demonstrate the correspondence between spectrum of $\sigma_{tot}$ and the eigen modes $\Psi$, we decompose the dipole moment of $j$-th atom in the basis of eigen modes: $\mathbf{d}^{(j)} = \sum_{n=1}^{N_{tot}} c_n \Psi_n^{(j)}$. After substitution of this decomposition into Eq.(S6), we obtain $\sigma_{tot} = \sum_{n=1}^{N_{tot}} \sigma_n$, where partial scattering cross-section $\sigma_n$, is defined as:

$$\sigma_n = \frac{4\pi k_0}{|\mathbf{E}_0(\mathbf{r})|^2}\sum_j \mathrm{Im}\, c_n \Psi_n^{(j)}\mathbf{E}_0^*(\mathbf{r}_j). \tag{S8}$$

Since we are considering highly non-homogeneous external fields, the denominator in Eq. (S7) strongly depends on the choice of a spatial point at which the excitation field is taken. Therefore, unlike the plane-wave excitation, the scattering cross-section in the case of the Bessel beams is defined up to a constant. To obtain some figure of merit, we normalize scattering cross-section of the system to cross-section $\sigma_0$ of a single atom positioned at maximum of longitudinal component of incident field $E_{0z}$.

During the calculations of scattering spectra shown at Fig. 6, we considered $\mathbf{E}_0(\mathbf{r})$ as a profile of vector Bessel beam in according with Eqs (5), (7-8) from Ref. [6]. We took parameters of right circular polarized beam ($s = \sigma = 1$) with orbital momenta $l = m = 7$ and $l = m = 9$, value of numerical aperture $NA = 1$, ratio of the pupil radius and the Gaussian beam waist $\beta = 0.5$.

[1] A. Asenjo-Garcia, M. Moreno-Cardoner, A. Albrecht, H. J. Kimble, and D. E. Chang, "Exponential Improvement in Photon Storage Fidelities Using Subradiance and ``Selective Radiance" in Atomic Arrays," *Phys. Rev. X*, vol. 7, no. 3, p. 031024, 2017.

[2] L. Novotny and B. Hecht, *Principles of Nano-Optics.* Cambridge, England, UK: Cambridge University Press, 2012.

[3] P. A. Belov and C. R. Simovski, "Homogenization of electromagnetic crystals formed by uniaxial resonant scatterers," *Phys. Rev. E*, vol. 72, no. 2, p. 026615, 2005.

[4] J. Wiersig, "Formation of long-lived, scarlike modes near avoided resonance crossings in optical microcavities," *Phys. Rev. Lett.*, vol. 97, p. 253901, Dec 2006.

[5] B. T. Draine, "The Discrete-Dipole Approximation and Its Application to Interstellar Graphite Grains," *Astrophys. J.*, vol. 333, p. 848, Oct. 1988.

[6] A. G. N. V. V. Kotlyar and S. S. Stafeev, "Exploiting the circular polarization of light to obtain a spiral energy flow at the subwavelength focus," *J. Opt. Soc. Am. B, JOSAB*, vol. 36, no. 10, pp. 2850–2855, 2019.
